# Free, open, quantitative and adaptable digital soil map data and database for Nigeria

**DOI:** 10.1016/j.dib.2020.105941

**Published:** 2020-06-27

**Authors:** Ugonna C. Nkwunonwo, Francis I. Okeke, Elijah S. Ebinne, Ndukwe E. Chiemelu

**Affiliations:** Department of Geoinformatics and Surveying, University of Nigeria, Nsukka, Nigeria

**Keywords:** Digital soil map, Nigeria, Land resources, Geomorphology, Soil information, Digital cartography, Geographic information system, Raster

## Abstract

Nigeria's digital soil map (DSM) and database is the most comprehensive and detailed source of quantitative information on the country's soil distribution. Digital cartography and geographic information system (GIS) operations were methods used in producing the DSM and database. We obtained analogue soil data in 8 hard copy maps (each at a displayable scale of 1: 650,000) from the Federal Department of Agricultural Land Resources (FDALR), which archived the result of Nigeria's reconnaissance soil survey. The survey started in 1985 and by 1990; it has completed the compilation of hard copy maps that outline Nigeria's major soil distribution. Our experimental design begins with electronic scanning of these paper maps. We set the scanning system to 500 dpi, creating high-resolution raster images, which were imported into ESRI ArcGIS software, for orthorectification by geo-referencing to WGS 1984 geographic coordinate system. We applied a spatial processing tool on the orthorectified images and created a geometrically-seamless mosaicked raster image for the soil data of the whole Nigeria. Using GIS on-screen digitization – with optimal snapping tolerance – we created vector polygons (spatial data) of soil components (mapping units). Finally, we coded the metadata (attributes) of Nigeria's soil distribution into Microsoft EXCEL spreadsheet, which we linked to the soil spatial data. The combined spatial and attribute soil data forms the soil database for Nigeria and provides, on-demand, vital soil information, such as thematic maps of soil characteristics. The department of Geoinformatics and surveying University of Nigeria, Enugu Campus (UNEC) is the major repository of Nigeria's DSM and database.

**Specifications Table**

**Table:** 

**Subject**	Environmental Sciences (General)
**Specific subject area**	Geographic Information System (GIS), Digital Soil Mapping
**Type of data**	Image Figure Digital maps (quantitative), Metadata (Attributes)
**How data were acquired**	GIS spatial analyses, map design and raster to vector conversion Instruments: ESRI ArcGIS 10.2.1 installed in a I TB HDD, 6GB RAM and 2.4 GHz CPU HP Desktop computer interphase with 21 inches flat screen Monitor.
**Data format**	Raw Vector shapefiles (.shp); Soil database, Raster images (Tiff, JPEG)
**Parameters for data collection**	Electronic scanning of hardcopy maps was set to 500 dpi to create high-resolution raster images. Georeferencing was targeted to WGS 1984. Optimal snapping tolerance was enabled to ensure accurate onscreen vectorization.
**Description of data collection**	Acquisition of analogue soil data from the Federal Department of Agricultural Land resources (DALR), Electronic scanning of the hard copy soil maps into raster images, orthorectification of the raster images, onscreen vectorization, creation of soil attribute and linking of these attribute data to soil spatial data.
**Data source location**	Department of Geoinformatics and Surveying, University of Nigeria Enugu, south-eastern region, Nigeria
**Data accessibility**	Only the static graphic maps are included in this article. The main digital data are hosted securely as Mendeley Data. Direct URL to data: https://data.mendeley.com/datasets/zmrt6k83wk/draft?a=d9a35c1e-c19b-4ddd-b34e-69674a8ceb18

**Value of the Data**

• Nigeria's DSM data and database represent a geo-spatial infrastructure, providing a quick, dynamic, quantitative, easily accessible and adaptable soil information platform for agricultural and land-related purposes in Nigeria. The DSM data solve the cartographic and qualitative limitations inherent in the 1990 soil data compilation, which makes it difficult to blend easily for updating and more scientific operations [1].
• Nigeria's DSM data and database are of primary value to those working on improving food security through soil suitability assessment, mechanised farming and selective cropping for sustainable land resources management [2]. Many soil scientists will find that the DSM data are a useful backdrop for extensive scientific analyses and pedometry involving remote sensing and field data verification [3]. Environmental scientists and researchers working on land degradation, heavy metal contaminations of the food chain, soil texture modelling, spatio-temporal characterisation of draught, site suitability analyses and natural hazards effects on land resources will find the DSM and database as indispensable tools [3,[4], [5], [6]].
• Considering its quantitative nature, Nigeria's DSM data and database allow users to both explore and interact with its derivatives [1]. By exploring the data, users can discover unique themes of soil characteristics which derive from the DSM data. By interacting with the spatial query tools and functions built into the DSM data and its database, users can find detailed answers relating to various spatial characteristics and identities of Nigeria's soil distribution. This can be useful for predicting the outcomes of spatially integrating of two or more soil characteristics.
• Evidence from [7], [8], [9] shows that world over global climate change endangers the Agro-ecological significance of most arable and grazing lands. Within the context of Nigeria, the DSM data and soil database provide suitable information to make the best use of soils, thus mediating the increasing demand for land resources – land tenure system – which often escalates tribal and economic tension within the country [2].
• Nigeria's DSM data offers flexibility for data sharing, data hosting on the internet, and integration into regional and global digital soil mapping programmes. As well as being able to provide thematic datasets based on soil properties in Nigeria, the digital soil database enhances the knowledge and value of Nigeria's soil for a myriad human and land-related needs. This will help to resolve complex environmental issues consistent within local and regional geological and geomorphological characteristics such as erosion, landslide and drought [3].

## 1 Data description

Nigeria's DSM data and soil database of Nigeria is a predictive or computer-assisted digital depiction of Nigeria's soil types, properties and spatial distribution. In this novel DSM data and soil database, secondary data in the form of compiled hard copies, derived from the 1985 reconnaissance soil survey are transformed into vectors and symbols encoded and held as data structures within the map layout. The DSM data and its database hold information relating to the Nigerian soil in geographically referenced numerical format. Thus, it delineates quantitatively and interactively the 58 soil components (soil mapping units), based on three ecological zones, twenty-four broad geomorphic units and parent materials in Nigeria. These soil mapping units are numbered from ‘1a’ to ‘24b’ and provides much quantitative information for environmental modelling and analyses using the Nigeria's DSM data and database (Refer to [1] for a full list of the mapping units that describes the Nigeria's soil distribution and their spatial distinctions).

**Fig. 1:**
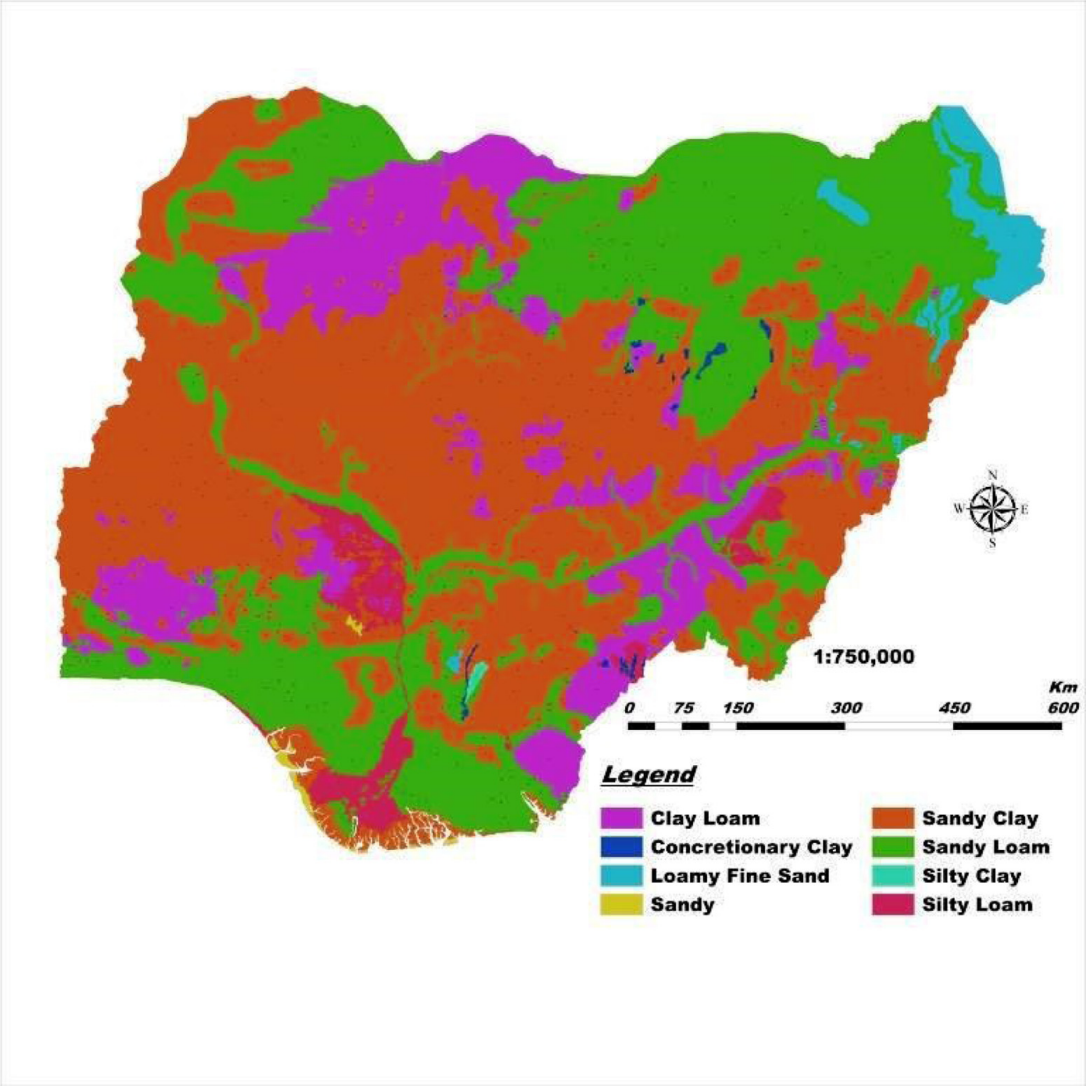
Soil texture map derived from Nigeria's DSM and database.

**Fig. 2:**
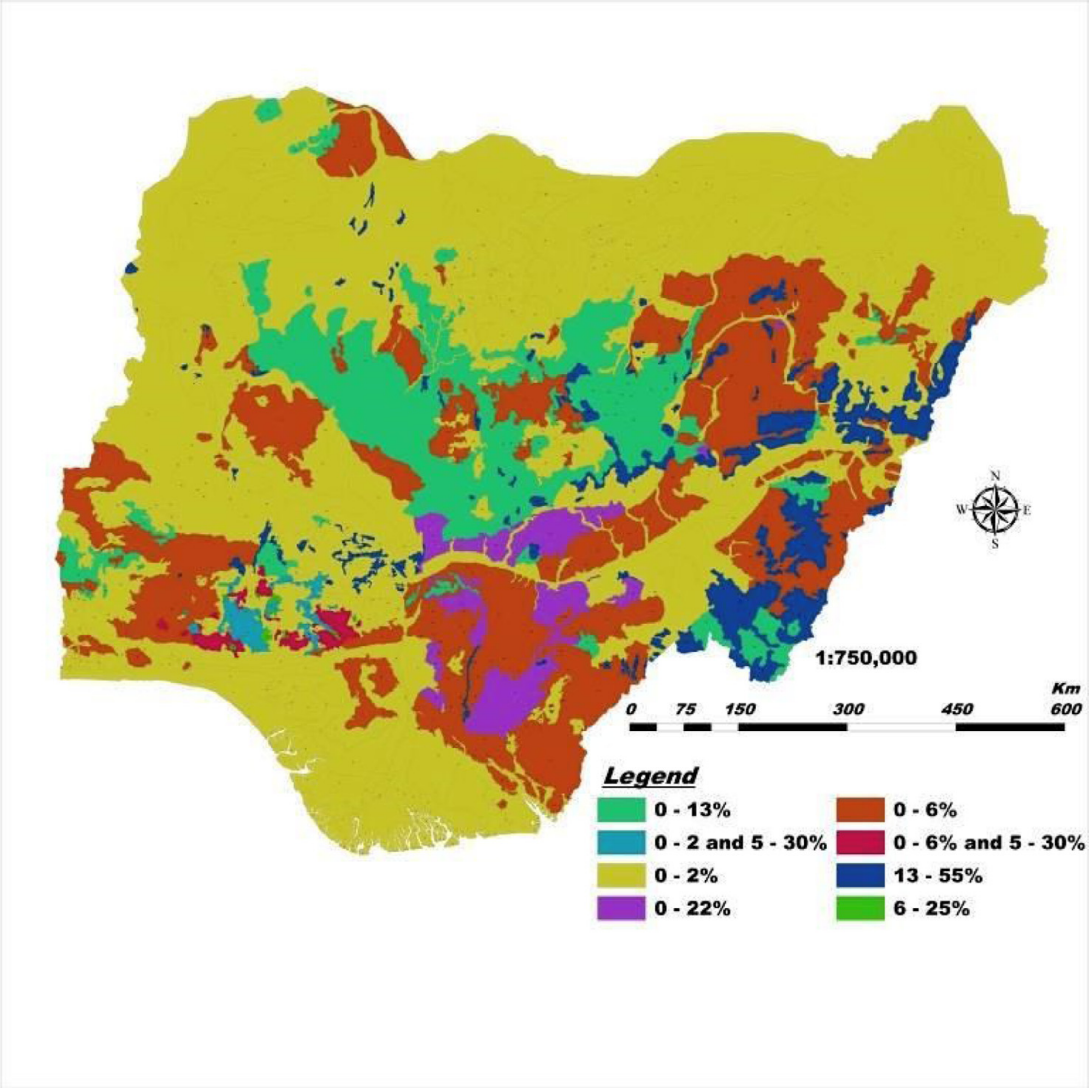
Soil slope map derived from Nigeria's DSM and database.

**Fig. 3:**
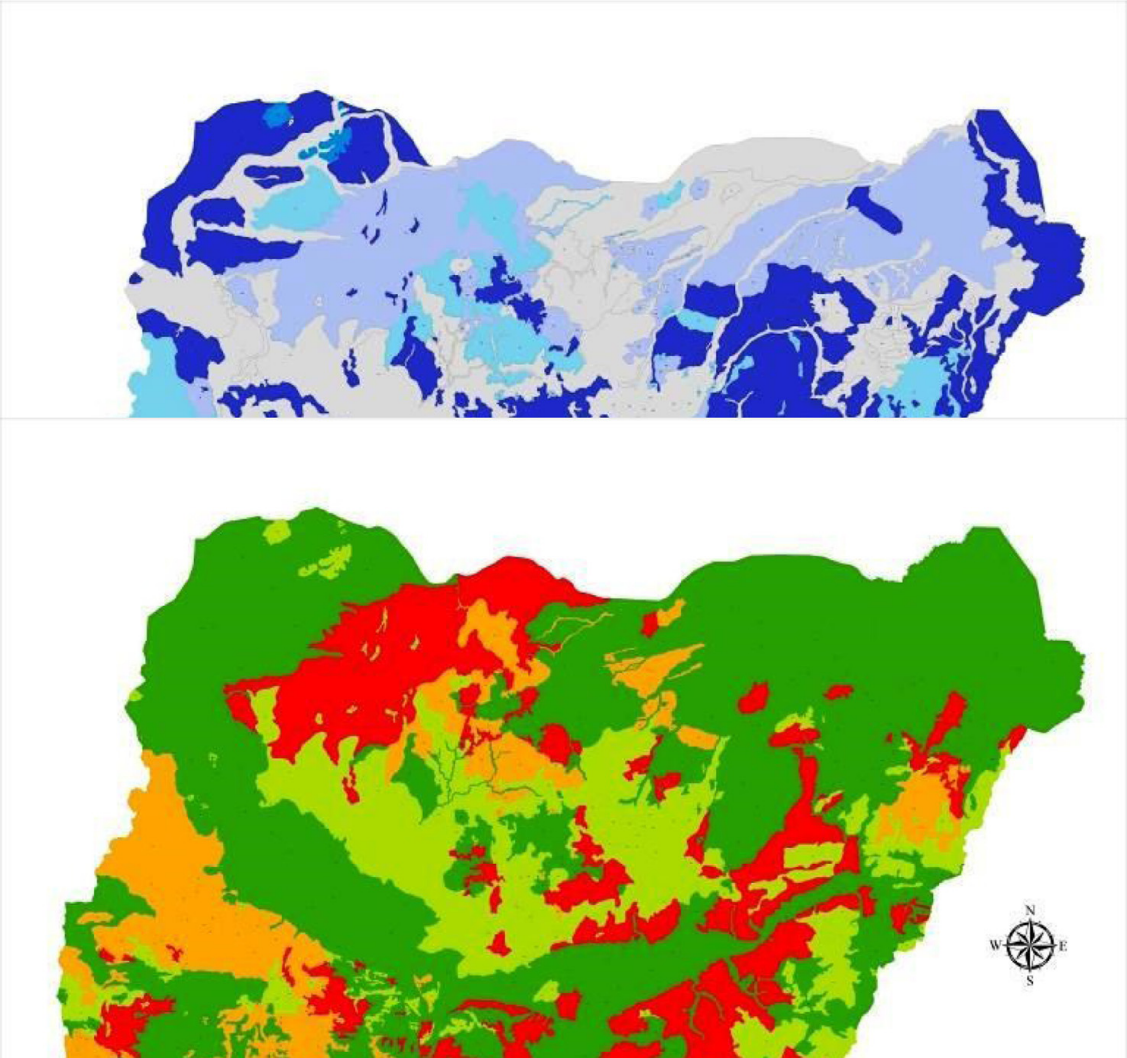
Soil drainage map derived from Nigeria's DSM and database.

**Fig. 4:**
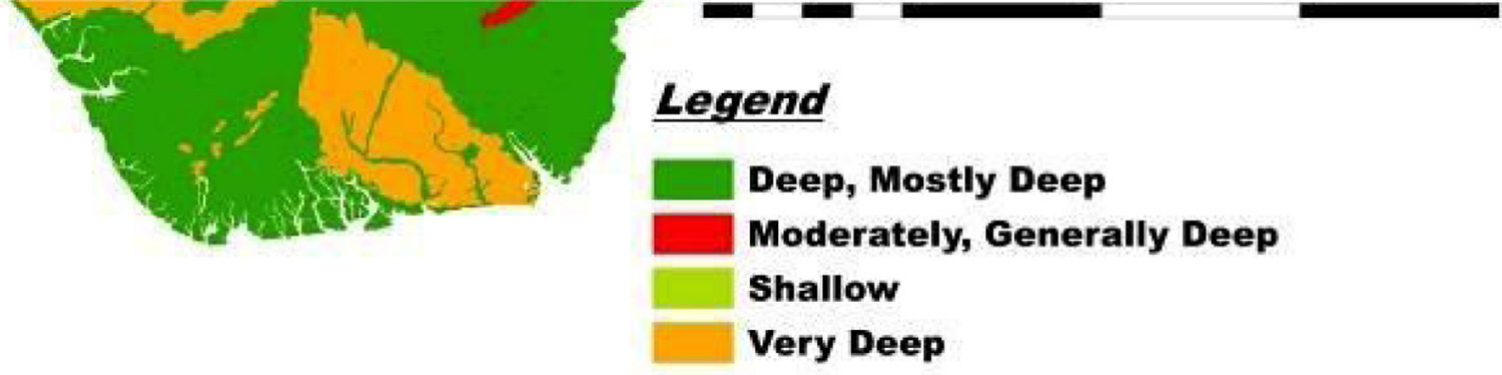
Soil depth map derived from Nigeria's DSM and database.

**Fig. 5:**
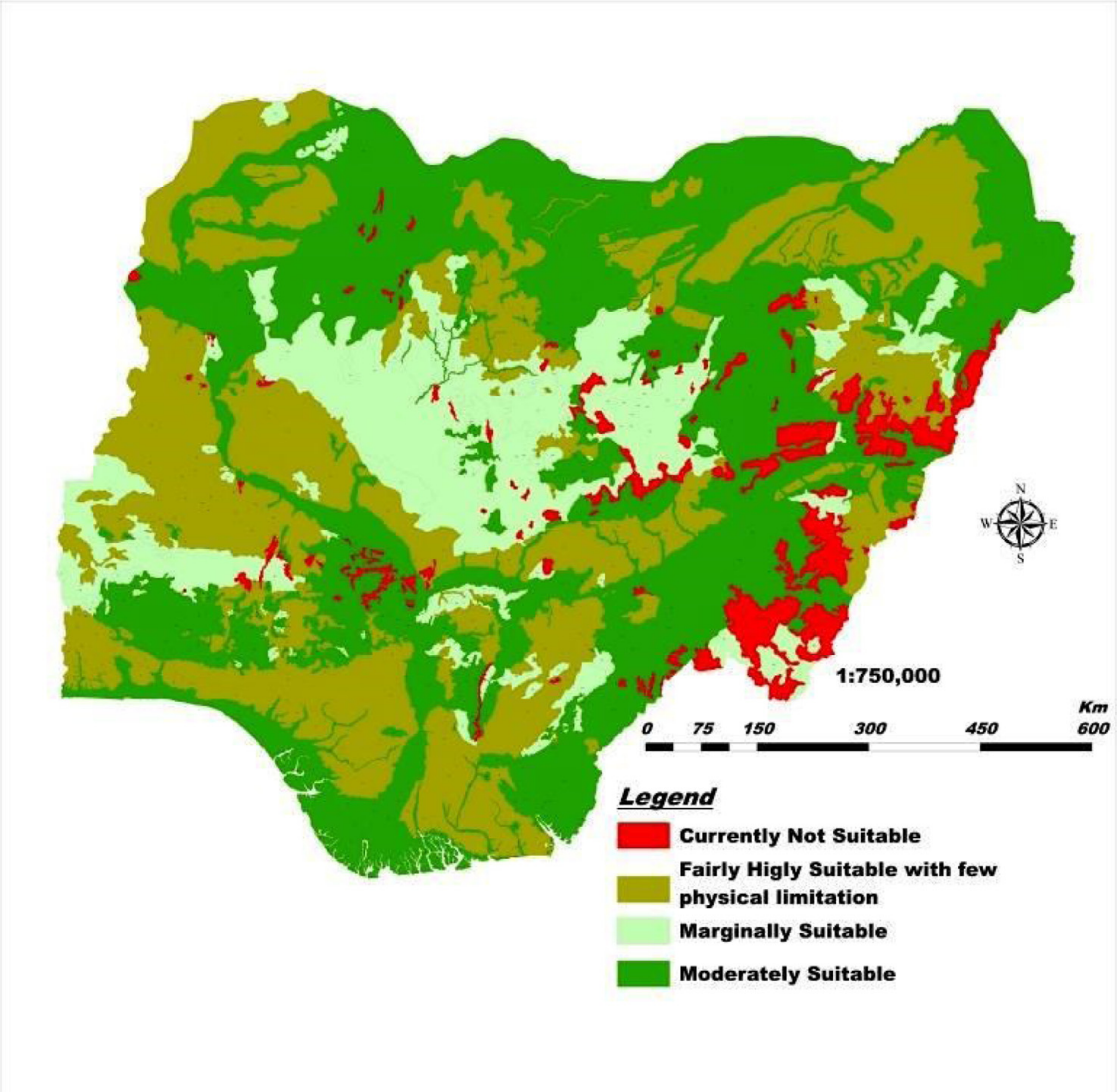
Soil suitability to mechanized farming map derived from Nigeria's DSM and database.

This DSM and its database have a highly unique legend with flexible scales, are easy to update with location-specific measure of accuracy, and guarantees data security. Users can quickly generate spatial soil information that provides ample solutions to the growing demand for high-resolution soil data. This solves the cartographic and qualitative limitations of the 1990 FDALR hard copy soil maps. The DSM incorporates queries and spatial analyses tools to generate thematic maps of basic soil properties within the context of Nigeria such as soil mapping unit, geology (basement complex, basalt, sandstone, Aeolian sandstone, shale, etcetera), soil classification (USDA and FAO), pH scales (acidic, basic, neutral, etcetera) and ecological zones (wetland, rainforest, savanna). In this article, we present Figs. 1–5 which are thematic maps derived from the Nigerian DSM data and database. Fig. 1 is the soil texture which describes in digital format the 8 soil texture categories in Nigeria. These are disparate combinations of sandy, clay, loam and silt. Fig. 2 is the digital representation of the soil slope. For Nigeria, this varies from 0.15% to 55%. Fig. 3 is the digital drainage map which shows that more areas within the country are well drained. Fig. 4, Fig. 5 represent the soil depth and soil suitability to mechanised farming. These are all digital representation of the actual geomorphological features within Nigeria. The DSM incorporates the capacity to select all areas in Nigeria with potential to support the growth of particular crops and vegetative cover such as yam and cassava.

## 2 Experimental design, materials and methods

**Fig. 6:**
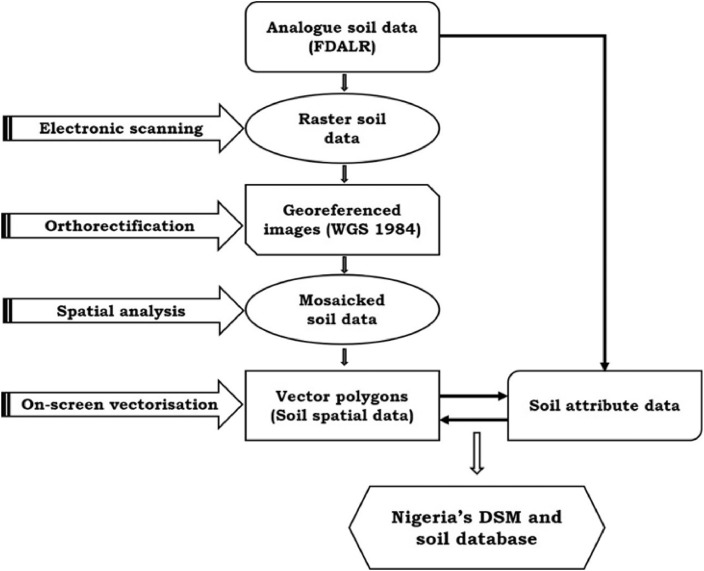
Experimental flow chart of the production of Nigeria's DSM and soil database.

We obtained analogue soil data in 8 hard copy maps (each at a displayable scale of 1: 650,000) from the Federal Department of Agricultural Land Resources (FDALR). These maps, along with the secondary information that describes the soil characteristics are the key primary input to the Nigerian DSM and soil database. These inputs were derived from the Nigeria's reconnaissance soil survey which was completed in 1990. Our experimental design begins with electronic scanning of these paper maps. We set the scanning system to 500 dpi, creating high-resolution raster images, which were imported into ESRI (Erath System Resource Institute) ArcGIS software, for orthorectification by geo-referencing to WGS 1984 geographic coordinate system. We applied ArcGIS spatial processing tool on the orthorectified images and created a geometrically-seamless mosaicked raster soil image for the whole Nigeria. Using the on-screen digitization – with optimal snapping tolerance – we created vector polygons (spatial data) of soil components (mapping units). Finally, we coded the metadata (attributes) of Nigeria's soil distribution into Microsoft EXCEL spreadsheet, which we linked to the soil spatial data. The table of soil characteristics description was then linked to the vector features to form the digital Nigeria soil database. Although ESRI ArcGIS is a proprietary software, its versatility, easy to use modes and abundance of toolsets made it a preferred software in the experimental design and development of Nigeria's DSM. Fig. 6 below shows the experimental flow chart for the production of Nigeria's DSM and soil database.

## Ethics Statement

Not applicable

## Declaration of Competing Interest

The authors declare that they have no known competing financial interests or personal relationships which have, or could be perceived to have, influenced the work reported in this article.
